# Dysfunctional Glymphatic System with Disrupted Aquaporin 4 Expression Pattern on Astrocytes Causes Bacterial Product Accumulation in the CSF during Pneumococcal Meningitis

**DOI:** 10.1128/mbio.01886-22

**Published:** 2022-08-29

**Authors:** Jaqueline S. Generoso, Sigrun Thorsdottir, Allan Collodel, Diogo Dominguini, Roberta R. E. Santo, Fabricia Petronilho, Tatiana Barichello, Federico Iovino

**Affiliations:** a Laboratory of Experimental Neurology, Graduate Program in Health Sciences, University of Southern Santa Catarina (UNESC), Criciúma, Santa Catarina, Brazil; b Department of Neuroscience, Karolinska Institute t, Biomedicum, Stockholm, Sweden; c Laboratory of Experimental Pathophysiology, Graduate Program in Health Sciences, University of Southern Santa Catarina (UNESC), Criciúma, Santa Catarina, Brazil; d Translational Psychiatry Program, Faillace Department of Psychiatry and Behavioral Sciences, McGovern Medical School, The University of Texas Health Science Center at Houston, Houston, Texas, USA; Carnegie Mellon University

**Keywords:** meningitis, *Streptococcus pneumoniae*, glymphatic system, neuroinflammation, neuronal damage, aquaporin 4, astrocytic end feet, blood-brain barrier (BBB)

## Abstract

Pneumococcal meningitis, inflammation of the meninges due to an infection of the Central Nervous System caused by Streptococcus pneumoniae (the pneumococcus), is the most common form of community-acquired bacterial meningitis globally. Aquaporin 4 (AQP4) water channels on astrocytic end feet regulate the solute transport of the glymphatic system, facilitating the exchange of compounds between the brain parenchyma and the cerebrospinal fluid (CSF), which is important for the clearance of waste away from the brain. Wistar rats, subjected to either pneumococcal meningitis or artificial CSF (sham control), received Evans blue-albumin (EBA) intracisternally. Overall, the meningitis group presented a significant impairment of the glymphatic system by retaining the EBA in the CSF compartments compared to the uninfected sham group. Our results clearly showed that during pneumococcal meningitis, the glymphatic system does not function because of a detachment of the astrocytic end feet from the blood-brain barrier (BBB) vascular endothelium, which leads to misplacement of AQP4 with the consequent loss of the AQP4 water channel's functionality.

## INTRODUCTION

Streptococcus pneumoniae (the pneumococcus) remains the most significant pathogen responsible for community-acquired bacterial meningitis, a life-threatening inflammation of the meninges surrounding the brain and spinal cord caused by a bacterial infection of the Central Nervous System (CNS) (1). Notably, S. pneumoniae is the most frequent pathogen associated with bacterial meningitis in children and adults (2, 3). In children who survive an episode of pneumococcal meningitis, persistent cognitive impairment in low-resource countries is estimated to occur in 4% to 41% (4). In adults who survived pneumococcal meningitis in high-resource countries, cognitive impairment is estimated to occur in 32% of survivors (4, 5). The inflammation triggered by the host immune response leads to the recruitment of peripheral immune cells into the brain and the cerebrospinal fluid (CSF) to eliminate invasive pathogens (6). The inflammatory mediators that are released in the brain during the infection include cytokines, chemokines, reactive oxygen and nitrogen species, and neurotoxic molecules that contribute to the activation of the microglial cells, initiating neuroinflammation (7–9). All of these waste products, including host-derived debris, the invading pathogen itself, and any secreted toxins, are released in the CSF and brain parenchyma and must be efficiently cleared from the brain to regain CNS homeostasis. The glymphatic system is a network of perivascular channels that run throughout the brain and enable the interchange of CSF and brain interstitial fluid (ISF), which surrounds the parenchymal cells of the brain. Following this fluid dynamics, the glymphatic system shunts CNS-derived molecules and immune cells from the CNS and meninges to the draining lymph nodes (10). When the fluid dynamics of the glymphatic system are disrupted, toxic waste accumulates in the brain, further exacerbating the inflammation and interfering with disease recovery. This study hypothesized that pneumococcal meningitis impaired the glymphatic system's functionality, decreasing neurotoxic waste clearance in the brain. To test our hypothesis, adult Wistar rats were subjected to pneumococcal meningitis or artificial CSF (aCSF) (sham control) and Evans blue-albumin (EBA), and the glymphatic system function in the brain and the peripheral circulatory system was analyzed. Significantly more EBA was retained in the brain, particularly in the CSF compartments, in the meningitis group. Intracisternal administration of pneumococci in the CSF progressively caused decreased drainage of CSF into the brain parenchyma and diminished fluid return into the peripheral circulation. This finding is in line with a study by Pavan and collaborators in which it was reported that pneumococcal meningitis induced with intracisternal administration of S. pneumoniae caused accumulation of a fluorescent tracer in the CSF compartment as a consequence of impaired functionality of the glymphatic system (11). In this study, we newly observed a significant accumulation of pneumolysin and pneumococcal capsule localized in the CSF compartments rather than in the brain parenchyma over time. Neuroinflammation and neuronal damage progressed over time in the brain due to the infection. Finally, contributing to the malfunctioning of the glymphatic system, we observed during bacterial meningitis a detachment of the astrocytic end feet from the blood-brain barrier (BBB) vascular endothelium, which leads to the misplacement of aquaporin 4 (AQP4) water channels, which causes the interruption of exchange of between the CSF and the brain interstitial space.

## RESULTS

### Impairment of glymphatic system's functionality during pneumococcal meningitis.

**FIG 1:**
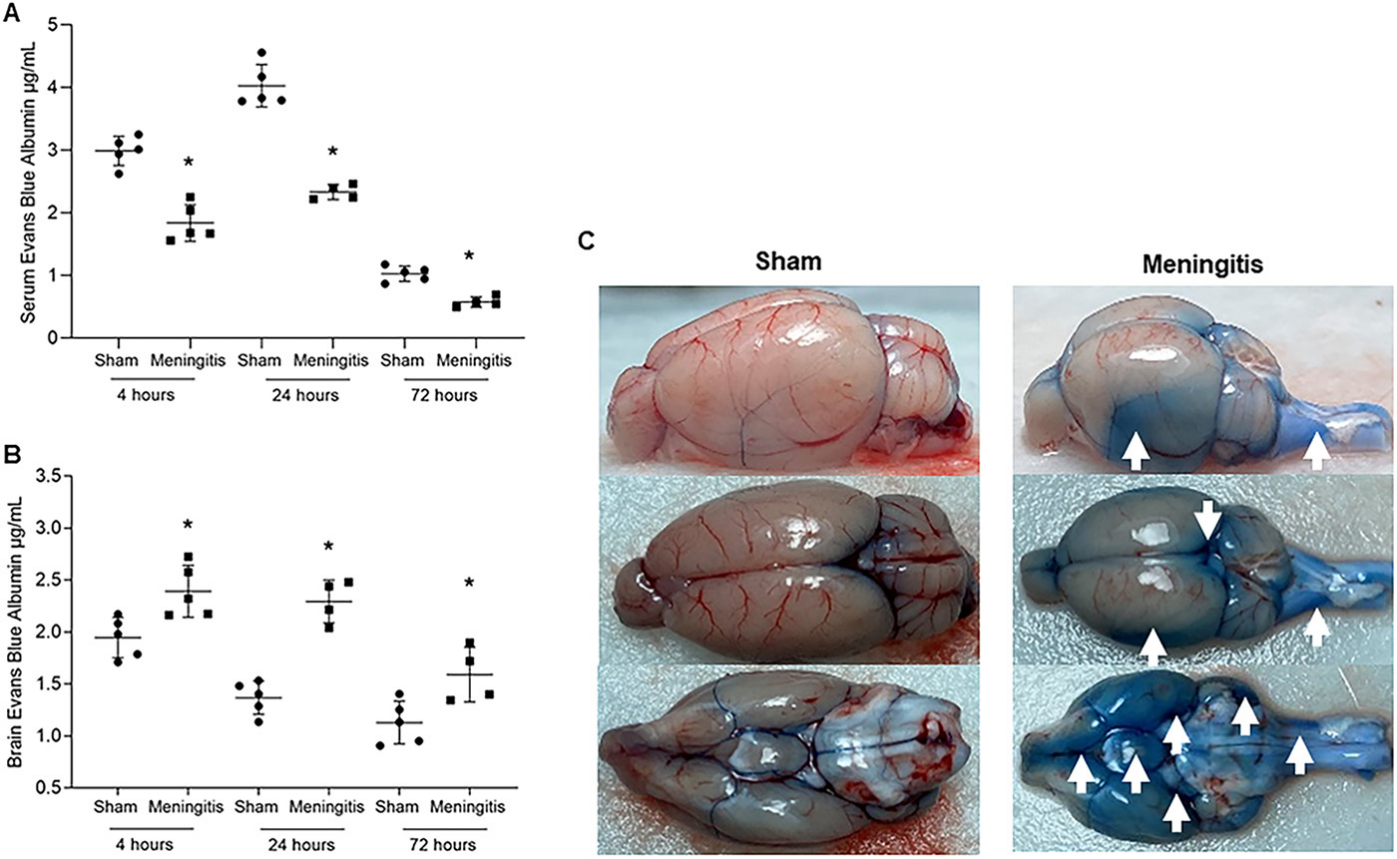
Demonstration of impaired glymphatic system's functionality during experimental pneumococcal meningitis *in vivo*. (A and B) Transit of EBA from cisterna magna to serum (A) and from cisterna magna to the brain (B) of adult Wistar rats at 4, 24, and 72 h after induction of pneumococcal meningitis. Data are shown as mean and standard deviation. Statistical significance is shown compared to the control group (*n* = 5). *, *P* < 0.05. (C) Retained EBA in the brains of the sham (noninfected) control and meningitis groups at the 4-h time point (the time point with higher EBA retention for both groups). Images of one brain/group are shown as representatives for both groups, and images have been taken from three different perspectives (side, top, and backwards). White arrows point toward the CSF compartments of the brain with high EBA retention.

Using an experimental meningitis rat model with intracisternal administration of EBA in combination with serotype 3 S. pneumoniae, we investigated the loss of functionality of the glymphatic system during CNS pneumococcal infection (see Fig. S1 in the supplemental material). We measured the EBA levels in the serum of animals subjected to pneumococcal meningitis. At 4, 24, and 72 h of infection, the meningitis group presented a decrease in the EBA levels in the serum compared with the sham control group, demonstrating that the animals presented impairment of the glymphatic system (Fig. 1A). We then evaluated whether the animals presented impairment of the glymphatic system's functionality by measuring the EBA content retained in the brains of animals subjected to pneumococcal meningitis. As a result, the EBA levels in the meningitis group's brains were significantly higher than those observed in the sham group at all three time points (Fig. 1B). Notably, in the brains of the rats in the meningitis group, EBA accumulation was detected all over the external layer of the subarachnoid space, as well as in the cerebellum, wherein the fourth ventricle CSF is produced, and in the spinal canal, where the CSF enters through the obex from the fourth ventricle (Fig. 1C).

10.1128/mbio.01886-22.1 FIG S1 Representation of EBA injection in the cisterna magna. The needle is inserted into the center of the cisterna magna, and it starts the injection of EBA using the microinjection syringe pump. The solute is drained from the cerebral parenchyma by the perivenous fluid to the peripheral lymph nodes and circulatory system. Download FIG S1, TIF file, 1.5 MB. Copyright © 2022 Generoso et al. 2022 Generoso et al. https://creativecommons.org/licenses/by/4.0/ This content is distributed under the terms of the Creative Commons Attribution 4.0 International license.

### Loss of astrocytic interaction with the BBB vascular endothelium and misplacement of AQP4 water channels.

**FIG 2:**
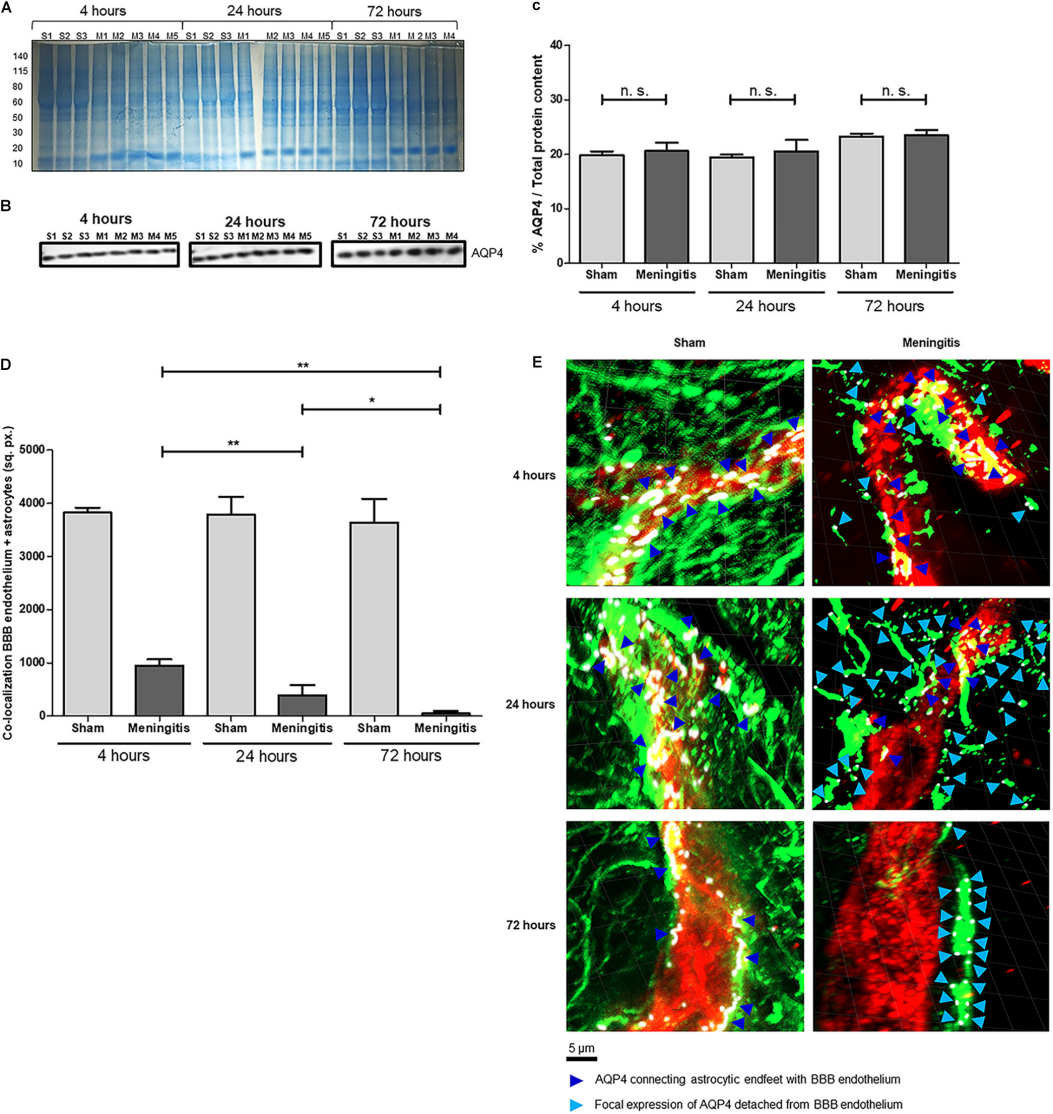
Loss of glymphatic system's functionality is not due to changes in AQP4 expression levels but to a misplacement of AQP4 water channels on astrocytic end feet. (A) The total protein content of brain homogenate samples was measured after Coomassie staining. S, sham (noninfected) control; M, meningitis. The numbers 1 to 5 refer to the rat number in the group (sham or meningitis) per time point; due to excessive bleeding, one brain (M5) from the 72-h time point was not used for this analysis. (B) Western blot detection of AQP4 in brain homogenate samples from sham and meningitis groups; since AQP4 is expressed by astrocytes independently from the infection, brain homogenates from three sham rats were analyzed; due to excessive bleeding; one brain (M5) from the 72-h time point was not used for this analysis. (C) The percentage of AQP4/total protein content was finally calculated using ImageJ, and data are shown as mean and standard deviation. n.s., nonsignificant. (D) Quantification of the area of colocalization (in square pixels [sq. px.]) between the BBB vascular endothelium (in red in panel E, as shown in Fig. S5) and the astrocytes (in green in panel E, as shown in Fig. S5) measured with ImageJ. Data are shown as mean and standard deviation. *, *P* < 0.05; **, *P* < 0.01. (E) Confocal microscopy analysis of BBB vascular endothelium (red), astrocytes (green), and AQP4 (purple, rendered in white using the imaging program ZEN lite). Dark blue arrows point toward AQP4 fluorescent signal connecting astrocytic end feet with BBB vascular endothelium, while light blue arrows point toward focal expression of AQP4 on astrocytes detached from the BBB endothelium. Four brain tissue sections from three sham control and four infected rats were analyzed per time point, and three images per section were taken. The displayed images are representative of each group. Plane images were angled 30° from the horizontal position on the *z* axis using Imaris.

After observing the EBA retention in the brain and the nondrainage of EBA in the serum in the meningitis group, we started investigating the interplay between astrocytes and the BBB. AQP4 is a water channel present on the end feet of perivascular astrocytes, which are in contact with the BBB, and AQP4 water channels regulate the solute transport of the glymphatic system (12–14). It was recently reported that AQP4 levels are not altered in the brain's cerebral cortex during pneumococcal meningitis (11). In line with these recent findings, our Western blot analysis using brain homogenates clearly showed that upon experimental pneumococcal meningitis, AQP4 protein levels are not altered, comparing brain tissue of the rats from the sham and meningitis groups over time (Fig. 2A to C). We then performed immunofluorescence microscopy analysis using brain tissue sections stained for the vascular endothelium of the BBB with Lycopersicon esculentum tomato lectin DyLight 594 (15) and astrocytes using anti-glial fibrillary acidic protein (anti-GFAP) antibody. Clear signs of astrogliosis were observed over time during pneumococcal meningitis infection. Reactive astrocytes, which retracted their cellular processes upon neuroinflammation, showed a progressive detachment of the astrocytic end feet from the BBB vascular endothelium (Fig. S2). The increased detachment of the astrocytic end feet from the BBB was further confirmed by the colocalization analysis performed by ImageJ, which showed that the connection between astrocytic end feet and the BBB is lost over time (Fig. 2D). To assess the misplacement of AQP4 water channels as a consequence of the astrocytic cellular process retraction, we also performed confocal microscopy analysis combined with three-dimensional (3D) reconstruction modeling. It is evident that during the course of pneumococcal infection, the progressive retraction of the astrocytic cellular processes causes the displacement of the AQP4, which loses its natural localization connecting the astrocytic end feet with the BBB vascular endothelium (Fig. 2D). Furthermore, while in the brain of sham control animals with a functioning glymphatic system, AQP4 expression is homogeneously expressed along the area between the astrocytic end feet and BBB vascular endothelium, in the brain of meningitis-affected rats in which the glymphatic system is malfunctioning, AQP4 expression is focal on astrocytic cells and far from any colocalization with the BBB endothelium (Fig. 2E). This misplacement of AQP4 water channels is likely the cause of the interruption of exchange between the CSF and the brain interstitial space during pneumococcal meningitis.

10.1128/mbio.01886-22.2 FIG S2 Immunofluorescence microscopy analysis of the detachment of astrocytic end feet from the BBB vascular endothelium when the glymphatic systems’ functionality is impaired. Shown are results from immunofluorescence microscopy analysis of BBB vascular endothelium (red) and astrocytes (green). Brain tissue sections from sham rats clearly show astrocytes in close proximity around the brain vasculature in support of the BBB; in contrast, brain tissue from meningitis-affected rats shows a progressive detachment over time of the astrocytic end feet from the vascular endothelium. The white double-arrows point towards the detaching astrocytic end feet. Four brain tissue sections from three sham rats and five (four for the 72-h time-point) meningitis rats were analyzed per time point, and 10 images per section were taken. The images displayed are representative of each group. Download FIG S2, TIF file, 0.9 MB. Copyright © 2022 Generoso et al. 2022 Generoso et al. https://creativecommons.org/licenses/by/4.0/ This content is distributed under the terms of the Creative Commons Attribution 4.0 International license.

10.1128/mbio.01886-22.5 FIG S5 Increase of the macrophage and microglia marker Iba1 during pneumococcal infection with a malfunctioning glymphatic system. (A) Western blot detection of Iba1 in brain homogenate samples of rats from noninfected sham control and meningitis groups. Since Iba1 is a microglial/macrophage marker present in the brain independently from the infection, brain homogenates from three sham rats were analyzed. Due to excessive bleeding, one brain (M5) from the 72-h time-point was not used for this analysis. (B) The percentage of Iba1/total protein content was finally calculated using ImageJ, and data are shown as mean and standard deviation. **, *P* < 0.01; n.s., nonsignificant. As a negative control, brain homogenate samples from three rats of the sham group per time point were analyzed. The numbers 1 to 5 refer to the rat number in the group (sham or meningitis) per time point. (The total protein content was measured after the Coomassie staining shown in Fig. 2A.) Download FIG S5, TIF file, 0.3 MB. Copyright © 2022 Generoso et al. 2022 Generoso et al. https://creativecommons.org/licenses/by/4.0/ This content is distributed under the terms of the Creative Commons Attribution 4.0 International license.

### Accumulation of bacterial components in the cerebrospinal fluid but not in the brain parenchyma.

**FIG 3:**
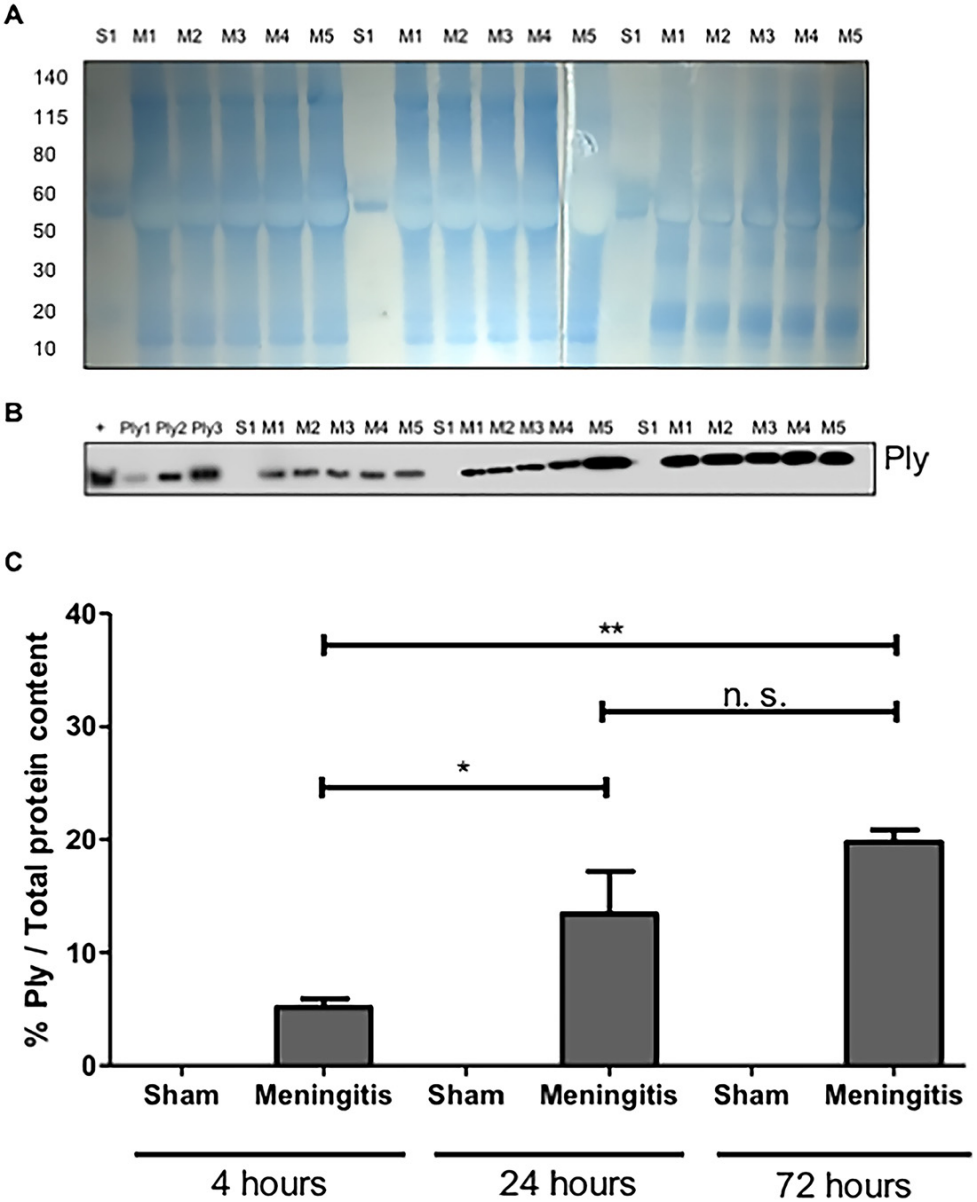
Accumulation of pneumococcal pneumolysin in the cerebrospinal fluid as a consequence of an impaired functionality of the glymphatic system. (A) The total protein content of cerebrospinal fluid (CSF) samples was first measured by Coomassie staining. S, sham (noninfected) control; M, meningitis. The numbers 1 to 5 refer to the rat number in the group (sham or meningitis) per time point. (B) The same volume of the same samples was then used for Western blot analysis for the detection of Ply; a lysate of serotype 3 S. pneumoniae was used as a positive control (+) together with three serial amounts of purified Ply (Ply1 at 0.0005 μg, Ply2 at 0.005 μg, and Ply3 at 0.05 μg in loaded volumes of 10 μL). (C) The percentage of Ply/total protein content was finally calculated using ImageJ. Data are shown as mean and standard deviation. *, *P* < 0,05; **, *P* < 0.01; n.s., nonsignificant since sham control rats were not infected with pneumococci and therefore did not have any Ply. As a negative control, one CSF sample from the sham group for each time point was analyzed, for a total of three sham samples analyzed.

**FIG 4:**
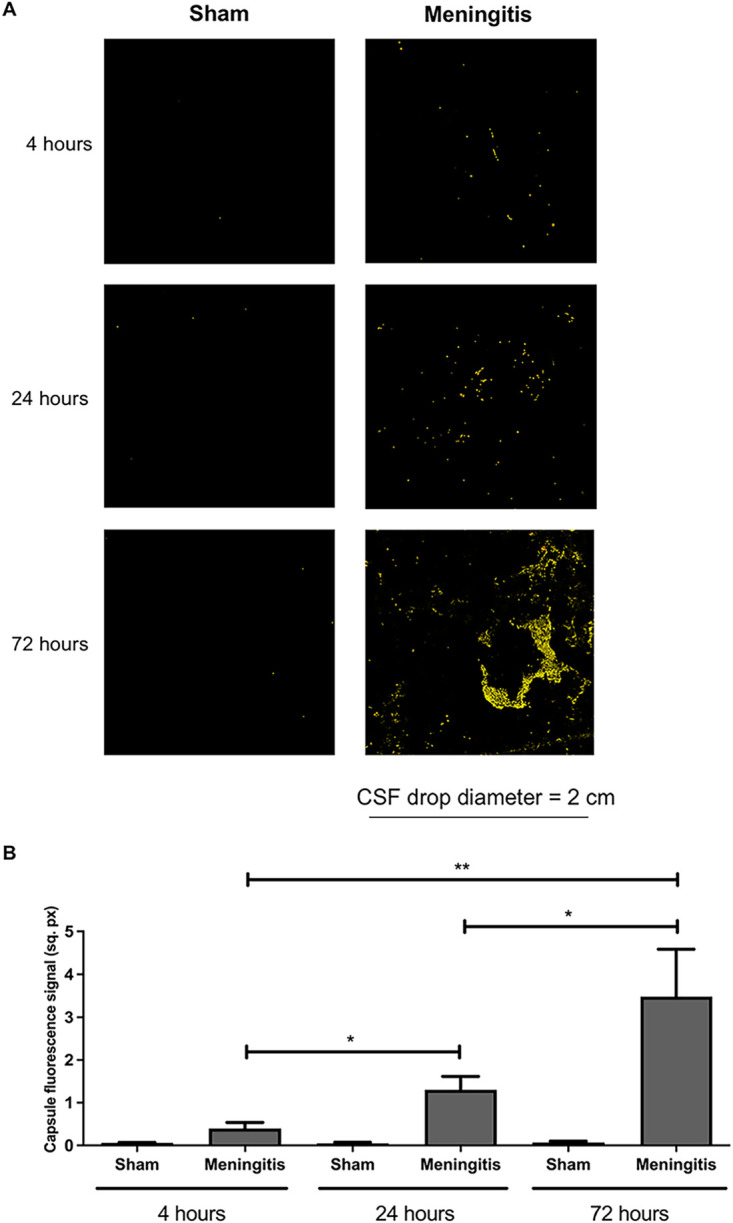
Accumulation of pneumococcal polysaccharide capsule in the CSF due to a malfunctioning glymphatic system during pneumococcal meningitis. (A) Ten-microliter CSF drops dried on microscope glass slides were stained for serotype 3 polysaccharide capsule (red). The surface area of the fluorescence images is within the yellow border (after the function “Edit selection” of ImageJ was applied to measure the surface area of the fluorescence signal) to enhance the contrast of the fluorescence signal. Per time point, CSF samples from three rats of the sham (noninfected) group were analyzed in order to have a broad assessment of the unspecific signal from noninfected CSF samples; one representative image is shown per group. (B) Quantification of the fluorescence signal of the pneumococcal capsule stained in Fig. 3A. Data are shown as mean and standard deviation. *, *P* < 0.05; **, *P* < 0.01.

**FIG 5:**
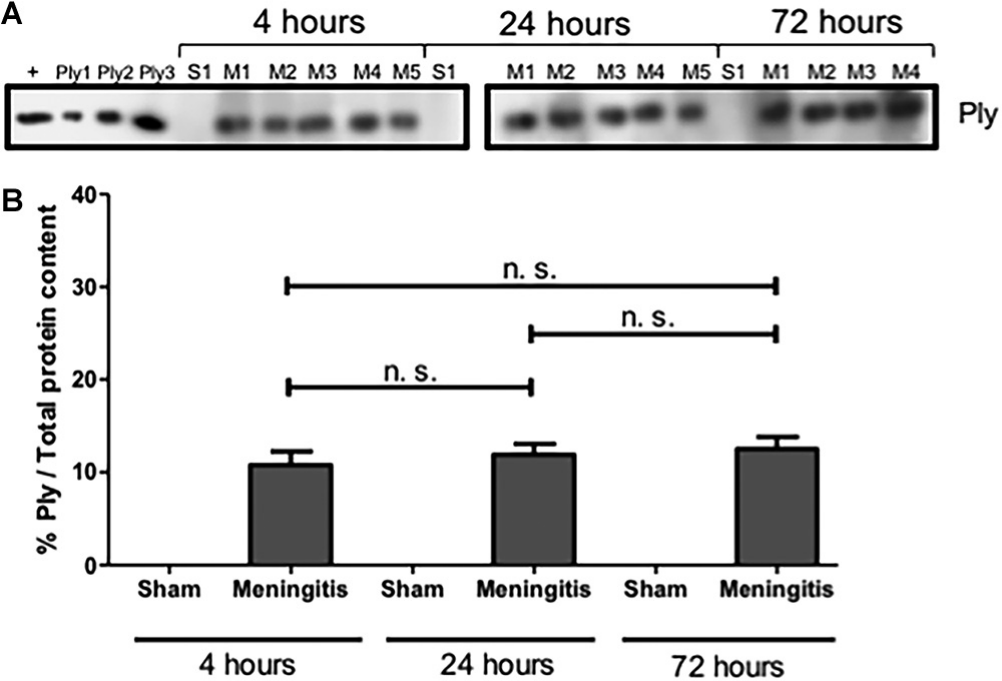
Presence but not accumulation of Ply in the brain homogenates of rats affected by pneumococcal meningitis. (A) Western blot analysis for the detection of Ply on brain homogenate samples. A lysate of serotype 3 S. pneumoniae was used as a positive control (+) together with three serial amounts of purified Ply (Ply1 at 0.0005 μg, Ply2 at 0.005 μg, and Ply3 at 0.05 μg in loaded volumes of 10 μL). As a negative control, brain homogenate samples from three rats of the sham (noninfected) group per time point were analyzed. (B) The percentage of Ply/total protein content was finally calculated using ImageJ. Data are shown as mean and standard deviation. n.s., nonsignificant. (The total protein content was measured after the Coomassie staining shown in Fig. 2A.)

**FIG 6:**
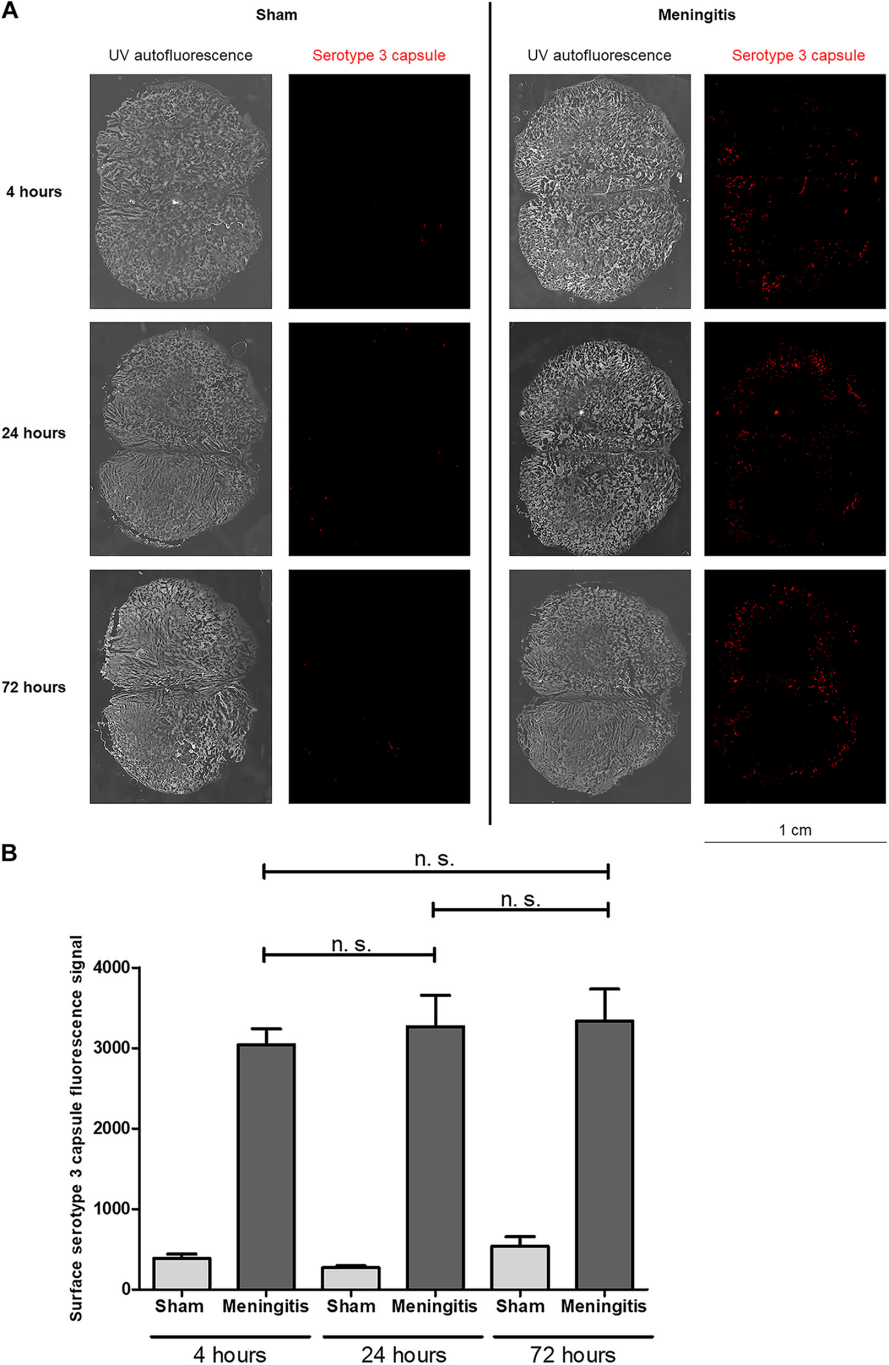
Presence but not accumulation of serotype 3 capsule in the brain parenchyma of rats with pneumococcal meningitis. (A) Brain tissue sections were stained for serotype 3 polysaccharide capsule (red), and the whole-tissue sections were imaged with autofluorescence under UV light. Per time point, tissue sections from one rat of the sham (noninfected) control group and three rats of the meningitis group with two sections per rat were analyzed in total. One representative image is shown per group (sham or meningitis) per time point. (B) Quantification of the fluorescence signal of the pneumococcal capsule stained in panel A. Data are shown as mean and standard deviation. n.s., nonsignificant.

To further study the loss of functionality of the glymphatic system during experimental pneumococcal meningitis, we evaluated the presence of pneumococci in the CSF of the rats subjected to pneumococcal meningitis to assess whether an accumulation of bacteria and/or bacterial products in the CSF would occur due to decreased CSF drainage into the brain parenchyma. Protein levels of pneumolysin (Ply), the main cytotoxin released by pneumococci, were measured by Western blotting, and a significant increase in Ply levels was detected in the CSF of the rats from 4 up to 72 h of pneumococcal infection (Fig. 3A to C; Fig. S3A). In addition, we also performed immunofluorescence microscopy analysis of the CSF samples, and we observed a significant increase in the fluorescent signal for serotype 3 polysaccharide capsule over time from 4 to 72 h (Fig. 4A and B); notably, the noninfected CSF samples did not show any fluorescence signal for pneumococcal capsule (Fig. 4A and B). Overall, over time, an evident accumulation of bacterial components was detected in the CSF of the rats during pneumococcal infection. In line with this accumulation of Ply and capsule, an increase in bacterial viability in the CSF was also detected (Table S1). On the other hand, we could not observe the same trend when we analyzed the presence of bacteria in the brain parenchyma. In fact, Western blot analysis using brain homogenates revealed that Ply expression levels did not increase over time (Fig. 5A and B; Fig. S3B), and immunofluorescence microscopy analysis of brain tissue sections showed no accumulation over time of the fluorescent signal of serotype 3 polysaccharide capsule in the brain parenchyma (Fig. 6A and B). To assess the capsule fluorescent signal accumulation over the brain tissue section, we first assessed whether the brain tissue sections from all rats analyzed from both sham control and meningitis groups had similar tissue surface areas. The whole-brain tissue sections were imaged using autofluorescence under UV light (Fig. 6A), and no significant differences were observed among brain tissue sections from different rats (Fig. S4). These results can be explained by the loss of functionality of the glymphatic system, which caused retained accumulation of bacterial components in the CSF. Furthermore, because of the reduced flow of CSF into brain tissue, bacterial components did not concentrate as much in the brain parenchyma as in the CSF. Taking all these findings together, loss of fluid drainage because of a malfunctioning glymphatic system leads to an accumulation of bacteria and bacterial products, as we previously observed for EBA, in the CSF compartments of the brain.

10.1128/mbio.01886-22.3 FIG S3 Putative concentrations of Ply in the CSF and brain parenchyma of rats affected by pneumococcal meningitis. Ply concentrations were calculated by measuring the band intensity of purified Ply at three different serial concentrations (Ply1 at 0.0005 μg, Ply2 at 0.005 μg, and Ply3 at 0.05 μg in loaded volumes of 10 μL) using ImageJ. Accordingly, the putative concentrations of Ply in the CSF (A) and brain homogenate samples (B) were calculated. Download FIG S3, TIF file, 0.2 MB. Copyright © 2022 Generoso et al. 2022 Generoso et al. https://creativecommons.org/licenses/by/4.0/ This content is distributed under the terms of the Creative Commons Attribution 4.0 International license.

10.1128/mbio.01886-22.4 FIG S4 Surface area of the brain tissue sections. Quantification of the surface area in square pixels (sq. px.) occupied by the brain tissue sections used for immunofluorescence microscopy (results shown in Fig. 6). For each rat, two brain sections were analyzed. Download FIG S4, TIF file, 1.3 MB. Copyright © 2022 Generoso et al. 2022 Generoso et al. https://creativecommons.org/licenses/by/4.0/ This content is distributed under the terms of the Creative Commons Attribution 4.0 International license.

10.1128/mbio.01886-22.7 TABLE S1 Clinical scores of the rats during experimental pneumococcal meningitis. Body weights were on average 216.6 ± 4.5 g for the 4-h group, 218 ± 6 g for the 24-h group, and 215.6 ± 3.8 g for the 72-h group. In the column “CFU/mL in CSF,” “AV” represents the average value for the meningitis group at each time point. (All animals in the sham control group had negative bacterial cultures.) Download Table S1, DOCX file, 0.01 MB. Copyright © 2022 Generoso et al. 2022 Generoso et al. https://creativecommons.org/licenses/by/4.0/ This content is distributed under the terms of the Creative Commons Attribution 4.0 International license.

### Increased neuroinflammation, neuronal cell damage, and impaired neurological functions upon pneumococcal meningitis with a malfunctioning glymphatic system.

**FIG 7:**
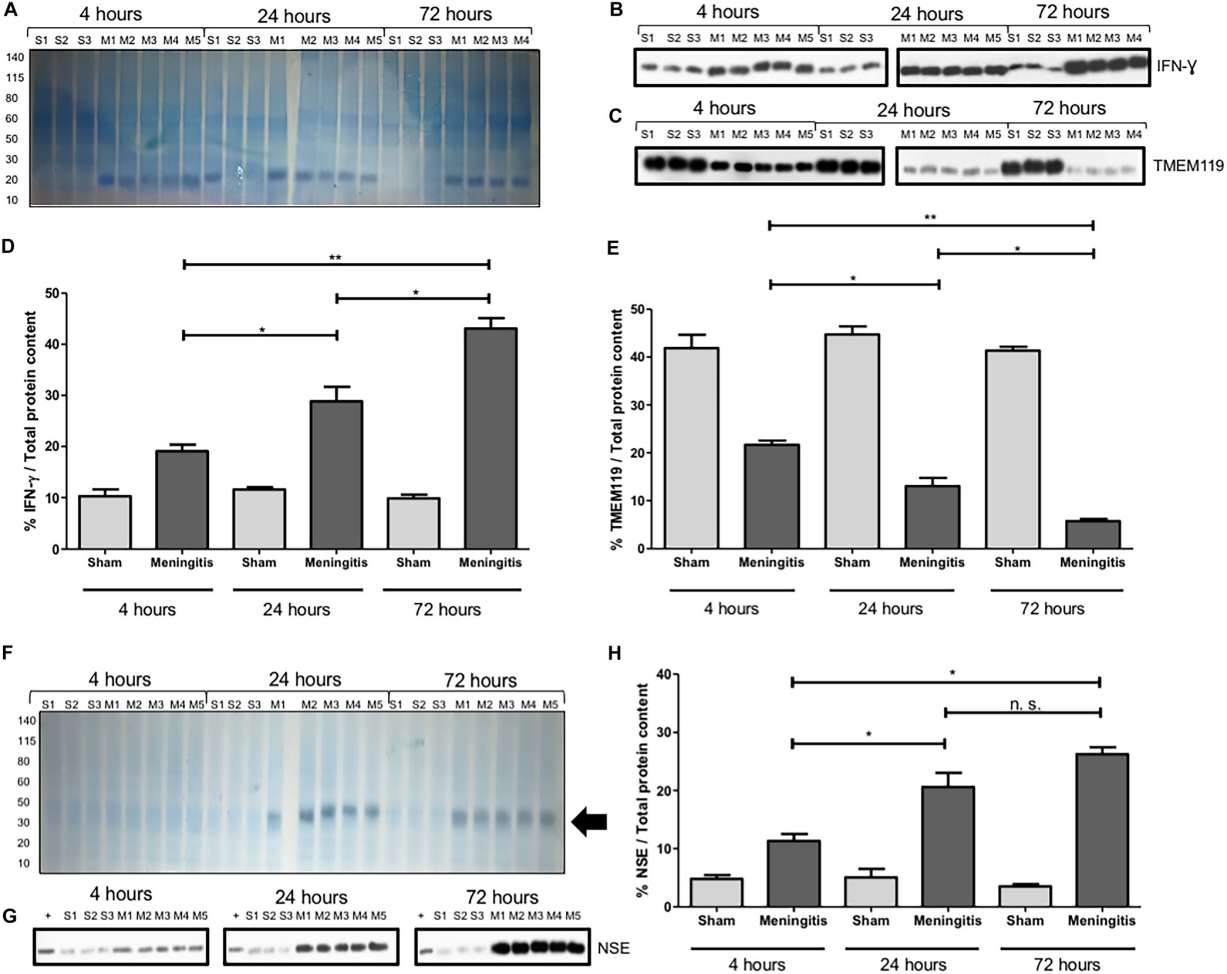
Neuroinflammation and neuronal damage increase over time during pneumococcal infection with a malfunctioning glymphatic system. (A) The total protein content of brain homogenate samples was measured after Coomassie staining. S, sham (noninfected) control; M, meningitis. The numbers 1 to 5 refer to the rat number in the group (sham or meningitis) per time point. (B and C) Western blot detection of IFN-γ (B) and TMEM119 (C) was performed using brain homogenate samples of rats from sham and meningitis groups. Since both TMEM119 and IFN-γ are, respectively, microglial and microglial/macrophage markers present in the brain independently from the infection, brain homogenates from three sham rats were analyzed. Due to excessive bleeding, one brain (M5) from the 72-h time point was not used for this analysis. (D and E) The percentages of IFN-γ/total protein content (D) and TMEM119/total protein content (E) were finally calculated using ImageJ. Data are shown as mean and standard deviation. *, *P* < 0.05; **, *P* < 0.01; n.s., nonsignificant. As a negative control, brain homogenate samples from three rats of the sham group per time point were analyzed. The numbers 1 to 5 refer to the rat number in the group (sham or meningitis) per time point. (The total protein content was measured after the Coomassie staining shown in Fig. 6A.) (F) The total protein content of serum samples was first measured after Coomassie staining. S, sham control; M, meningitis. The numbers 1 to 5 refer to the rat number in the group (sham or meningitis) per time point, and the black arrow points toward an enhanced intensity of the Coomassie staining around the molecular weight of NSE (around 40 kDa), which is particularly evident for the meningitis group at 24 and 72 h. (G) The same volume of the same samples was then used for Western blot analysis for the detection of NSE. A lysate of differentiated neurons from SH-SY5Y cells was used as a positive control (+). (H) The percentage of NSE/total protein content was finally calculated using ImageJ, and data are shown as mean and standard deviation. *, *P* < 0.05; n.s., nonsignificant.

**FIG 8:**
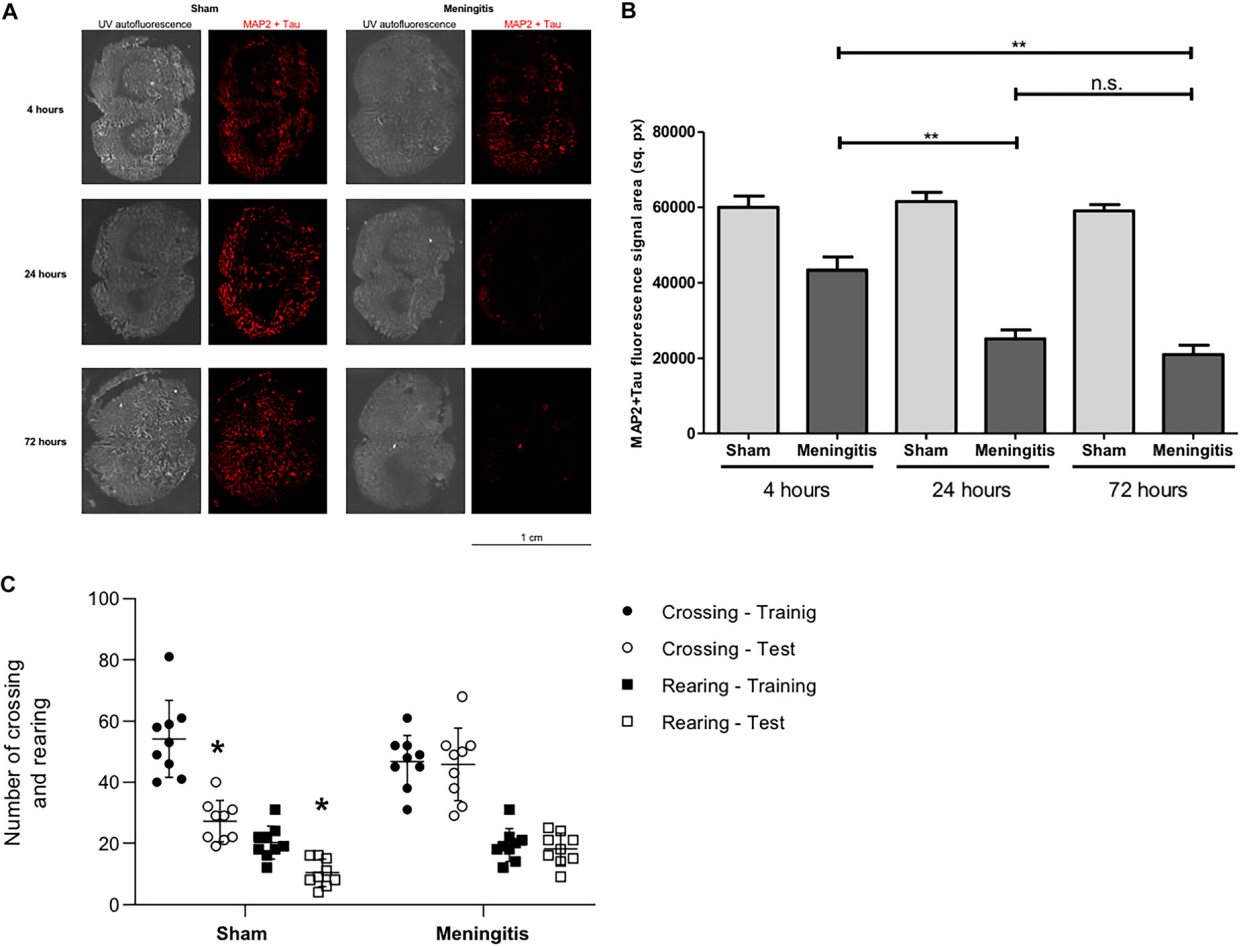
Loss of neuronal synaptic connections and consequent impairment of neurological functions during pneumococcal meningitis with a malfunctioning glymphatic system. (A) Brain tissue sections were stained for a combination of MAP2 and Tau proteins (red), and the whole-tissue sections were imaged with autofluorescence under UV light. Per time point, tissue sections from three rats of the sham (noninfected) group and three rats of the meningitis group with two sections per rat were analyzed in total. One representative image is shown per group (sham or meningitis) per time point. (B) Quantification of the fluorescence signal of MAP2+Tau stained in Fig. 5A. Data are shown as mean and standard deviation. **, *P* < 0.01; n.s., nonsignificant. (C) Open-field task 10 days after pneumococcal meningitis induction. Data from the open field task were reported as the mean and standard error of mean values and analyzed by paired Student's *t* tests (*n* = 9 animals per group). *, *P* < 0.05 versus training session.

As a consequence of an accumulation of bacteria and bacterial components in the CSF, we analyzed the consequent neuroinflammatory status in the brain. Using brain homogenates, we analyzed by Western blotting the protein levels of ionized calcium-binding adaptor molecule 1 (Iba1), microglia, and macrophage-specific marker (8). A significant increase in Iba1 levels was observed in the brains of the rats at 24 and 72 h compared to the 4-h time point of infection (Fig. S5A and B). This result points toward both activation of microglia in the brain and increased infiltration of macrophages into the brain over the course of the intracisternal pneumococcal infection. To further confirm the enhanced inflammatory process during pneumococcal meningitis with an impaired glymphatic system's functionality, we also measured by Western blot analysis the levels of interferon gamma (IFN-γ), a cytokine with a critical role in potentiating the proinflammatory signaling by priming macrophages and microglia during infections (16, 17). We observed a prominent increase of IFN-γ in the brain homogenates from 4 up to 72 h of pneumococcal infection (Fig. 7A and B). IFN-γ can also be secreted by activated microglia upon neuroinflammation and brain infections, as previously described (18, 19). Therefore, to assess a local neuroinflammatory response specific to microglia, we also measured by Western blotting the expression of the microglial-specific marker TMEM119 in the brain tissue of the rats at different time points of pneumococcal meningitis. It was recently described that TMEM119 is downregulated in microglia during neuroinflammation and brain disease (20–24). In line with what was reported in the literature, we observed a consistent decrease of TMEM119 expression in brain homogenates from 4 to 72 h of pneumococcal infection (Fig. 7A and C). Quantification analysis of the protein band intensities confirmed the significant increase of IFN-γ (Fig. 7D) and the significant decrease of TMEM119 (Fig. 7E) from 4 to up to 72 h of pneumococcal infection. Moreover, we have also investigated the degree of neuronal damage upon neuroinflammation. Damaged neurons release neuron-specific enolase (NSE), and detection in the blood is often performed in a clinical setting to quantitatively assess the neuronal damage during brain injuries, such as neuroblastoma in newborns, meningitis, and encephalitis (25–29). We analyzed the expression levels of NSE in the serum of the rats from the sham and meningitis groups, and a significant increase in NSE expression levels was detected in the serum of the rats from 4 up to 72 h of pneumococcal infection (Fig. 7F to H), indicating that enhanced neuronal damage has occurred in parallel with the increase of neuroinflammation in the case of an impaired glymphatic system's functionality during pneumococcal meningitis. To further confirm this, we performed immunofluorescence microscopy analysis using brain tissue sections and stained for microtubule-associated protein 2 (MAP2) and Tau, respectively, expressed mainly on neuronal dendrites and axons (30, 31), in order to semiquantify the amount of neuronal synaptic connections. We quantified the fluorescent signal of MAP2 and Tau proteins over the whole area of the brain tissue sections, considering that we had previously assessed that the surface areas of the brain tissue sections do not vary significantly between rats from the sham and meningitis groups (Fig. S4). The brain tissue sections of rats subjected to pneumococcal meningitis showed a significant decrease of MAP2 and Tau fluorescent signals over time, indicating a reduction of neuronal synaptic connections due to neuronal damage (Fig. 8A and B). To investigate further this neuronal cell damage caused by pneumococcal meningitis with an impaired glymphatic system's functionality, we performed open-field behavioral tests comparing the sham and meningitis groups. Since behavioral tests require longer times, new animal experiments were conducted in which new Wistar rats were subjected to the same infection with serotype 3 S. pneumoniae through intracisternal injection and then treated with antibiotic in order for the animals to sustain an infection for 10 days. At 10 days, the animals were subjected to the open-field task to investigate habituation memory after meningitis induction in rats with a proven impaired glymphatic system. In the sham group, we observed significant differences between training and testing sessions, as measured by the number of crossings and rearings. On the other hand, in the meningitis group, we did not observe any difference between training and testing sessions, indicating a consistent impairment of habituation memory (Fig. 8C).

## DISCUSSION

The BBB in the CNS allows the passage of O_2_, CO_2_, and glucose to satisfy the demand for brain cell metabolism but prevents unregulated entry of protein and fluid into the brain (32). However, the brain's interstitial space still receives soluble waste products due to its elevated metabolism (33). The glymphatic system maintains the balance by supporting the delivery of fluid and interstitial solutes, including immune cells and macromolecules, from the brain to the CSF egress routes to draining in the lymph nodes (34). The glymphatic dysfunction is characterized by a reduced exchange between CSF and interstitial fluid (ISF), leading to an accumulation of waste products. Many diseases have been associated with glymphatic system dysfunction, such as Alzheimer's disease, Parkinson's disease, and other neurodegenerative diseases (35–37). The BBB transport and glymphatic clearances are interdependent mechanisms, and these mechanisms’ dysfunction can make solute clearance difficult (37). BBB permeability or disruption facilitates the migration of peripheral immune cells and inflammatory mediators into the CSF, increasing the production of cytokines, chemokines, reactive oxygen and nitrogen species, and neurotoxic molecules that contribute to glial cell activation and consequent neuroinflammation (6–9). Preclinical studies of bacterial meningitis have demonstrated that BBB breakdown is associated with cognitive impairment (38, 39), and when proteolytic enzymes like matrix metalloproteinases (MMPs) were blocked, cognitive dysfunction could be prevented (40, 41).

Regarding BBB integrity during pneumococcal meningitis, it was also reported that blood-borne S. pneumoniae trafficking through the BBB does not cause significant disruption of the BBB vascular endothelium: in fact, VE-cadherin endothelial tight junctions have not been seriously compromised during the transmigration of bacteria across the BBB endothelium using a bacteremia-derived meningitis mouse model (15, 42). On the other hand, infiltration of peripheral immune cells into the brain can alter BBB endothelial integrity (43). However, it is known that leukocyte trafficking into the brain can occur via the glia limitans; leukocyte diapedesis requires accurate ligation mechanisms of adhesion molecules, selectins, and integrins, which promote the infiltration of leukocytes from the blood into the brain without disrupting the BBB endothelium (44). We believe that through retention, EBA, like other waste components and also toxic bacterial material, would still accumulate in the CSF compartments even in the case of a leaky BBB because EBA and pneumococci were injected directly into the CSF compartment, bypassing the passage through the BBB. This study evaluates the glymphatic function in an animal model of pneumococcal meningitis. The meningitis group presented accumulation of the EBA in the brain compared with the control group, demonstrating that the glymphatic function was affected by meningitis. The EBA was not drained adequately from the CSF to the lymphatic nodes, causing it to accumulate in the brains of the animals. To confirm this result, we also evaluate the EBA levels in the bloodstream of the animals. The control group presented an increase in EBA levels in the serum compared with the meningitis group, demonstrating that the meningitis group was unable to drain the EBA from the CSF to the bloodstream. Because of the loss of solute drainage between the CSF and the brain parenchyma, most of the pneumococcal components remained accumulated in the CSF compartments of the brain. Notably, a direct consequence of a malfunctioning glymphatic system is in fact brain edema, which is the buildup of fluid in the brain (45).

Neuronal injury is frequently observed upon neuroinflammation. Damage to neurons can be caused both by direct interaction of the bacteria or bacterial components, like pneumolysin, with neurons (46) and by the damaging effect of proinflammatory compounds released during the neuroinflammation process (47). In line with this, associated with a loss of glymphatic system functionality during pneumococcal meningitis pathogenesis, we showed that neuroinflammation and neuronal injury increase over time during the infection. Therefore, our data strongly suggest that glymphatic system dysfunction could be another factor associated with long-term cognitive impairment in meningitis survivors. Bacterial meningitis survivors present an increased risk of triggering of neurocognitive impairment. In a children's birth cohort, meningitis in early life was associated with neurocognitive, educational, and psychological difficulties during childhood and early adolescence (48). Another study also demonstrated that meningitis during childhood increases the risk of schizophrenia in adulthood (49). In adults, neurologic sequelae of pneumococcal meningitis in high-resource countries presented rates of 32% cognitive impairment, 4% hydrocephalus, 31% seizures, 22 to 69% hearing loss, and 11 to 36% focal deficits (4). The connections between BBB disruption, glymphatic dysfunction, and cognitive impairment in meningitis survivors could be a new avenue to investigate mechanisms to prevent these events and reestablish everyday function for patients still suffering dysfunction after bacterial meningitis.

AQP4 water channels are a fundamental component of the glymphatic system, facilitating solute transport from the perivascular subarachnoid space and the brain parenchyma (12–14). Upregulation of AQP4 expression levels was previously described upon neuroinflammation in neurodegenerative diseases, such as Alzheimer's disease (50) and multiple sclerosis (51). On the other hand, Pavan and collaborators have recently observed that upon neuroinflammation caused by a pneumococcal infection in the CNS, AQP4 expression levels are not significantly altered compared to those in a noninfected situation. Our results align with those of Pavan and collaborators (11), as we did not observe increased AQP4 expression levels in the brains of the meningitis-affected rats compared to the brains of the sham control rats. Significantly, what affects the solute transport of the glymphatic system during pneumococcal meningitis is not the altered AQP4 expression, but rather the disruption of the AQP4 water channels due to a detachment of the astrocytic end feet from the BBB. This cellular process is a straightforward consequence of the morphological changes of astrocytes that, during neuroinflammation, undergo astrogliosis, in which the cell body becomes rounder and more prominent, and the cellular processes become thicker and shorter (52, 53). The shortening of the astrocytic cellular processes causes the detachment of the astrocytic end feet from the BBB vascular endothelium. Such detachment causes the misplacement and consequent loss of function of the AQP4 water channels, which usually are present across the interspace between the BBB and the astrocytic end feet.

In conclusion, in this study we showed that during experimental pneumococcal meningitis, a loss of functionality of the glymphatic system occurs with a consequent accumulation of pneumococcal components, such as the cytotoxin Ply and the bacterial polysaccharide capsule used in this study as markers for the presence of S. pneumoniae, in the perivascular subarachnoid space occupied by CSF. Such accumulation of bacteria leads to a dramatic increase in neuroinflammation and neuronal injury; therefore, the loss of glymphatic system's functionality can play a crucial role in determining the neurological sequelae that often occur in patients who survive bacterial meningitis (4, 46), as shown by our results proving severe neuronal damage and impaired neurological functions when the glymphatic system is malfunctioning during pneumococcal meningitis (see Fig. S6 in the supplemental material). Astrocytes are a fundamental component of support for the BBB, with astrocytic end feet in close contact with the BBB vascular endothelium (54). Here, we reported for the first time that impairment of solute transport between the perivascular subarachnoid space and the brain parenchyma occurs when a detachment of astrocytic end feet from the BBB occurs, with consequent misplacement and likely loss of the physiological function of AQP4 water channels of solute transport within the glymphatic system.

10.1128/mbio.01886-22.6 FIG S6 Pathophysiology of a malfunctioning glymphatic system during pneumococcal meningitis. During pneumococcal meningitis, a loss of functionality of the glymphatic system occurs with consequent accumulation of pneumococci and pneumococcal components in the perivascular subarachnoid space occupied by CSF. Such accumulation of bacteria and bacterial toxic components leads to an increase in microglia activation, neuroinflammation, and neuronal damage. Upon neuroinflammation, the consequent astrogliosis causes a retraction of astrocytic cellular processes, which leads to a detachment of astrocytic end feet from the BBB vascular endothelium with consequent misplacement of AQP4; this AQP4 misplacement causes the loss of the physiological function of AQP4 water channels of solute transport within the glymphatic system. Download FIG S6, TIF file, 2.3 MB. Copyright © 2022 Generoso et al. 2022 Generoso et al. https://creativecommons.org/licenses/by/4.0/ This content is distributed under the terms of the Creative Commons Attribution 4.0 International license.

## MATERIALS AND METHODS

### Infecting organisms to induce meningitis.

Serotype 3 S. pneumoniae cells were cultured in 5 mL of Todd-Hewitt broth, diluted in fresh medium, and then grown to the logarithmic phase. The culture was centrifuged for 10 min at 1,200 rpm and resuspended in sterile saline to a concentration of 5 × 10^9^ CFU (41).

### Animal model of meningitis.

Experiments with Wistar rats were approved by the Animal Care and Experimentation Committee of UNESC 93/2019, Brazil and performed following the National Institutes of Health *Guide for the Care and Use of Laboratory Animals* (55). Male Wistar rats (8 weeks old, body weight of 200 to 250 g), obtained from our breeding colony and used for the experiments, were housed on a 12-h light/dark cycle, at a temperature of 23°C ± 1°C, with food and water always available *ad libitum*. Scoring of the clinical symptoms during pneumococcal infection was performed according to the research protocol approved by the Animal Care and Experimentation Committee of UNESC 93/2019, Brazil, and following the *Guide for the Care and Use of Laboratory Animals* (55) (Table S1). The animals were anesthetized with intraperitoneal (i.p.) administration of ketamine (6.6 mg/kg) and xylazine (0.3 mg/kg) (56–59). The animals were infected intracisternally with 10 μL of the inoculum containing 5 × 10^9^ CFU/mL of living S. pneumoniae; the sham control animals were injected with 10 μL of artificial cerebrospinal fluid (aCSF). The meningitis group (*n* = 5) and the control group (*n* = 5) received fluid replacement after bacterial induction. Meningitis was confirmed by incubating a quantitative culture of 5 μL of CSF at 37°C with 5% CO_2_ on sheep blood agar (60).

The same pneumococcal meningitis experiment was repeated without the use of EBA, and the time points of 4, 24, and 72 h were maintained; blood and brains were collected at the end of each time point to perform *ex vivo* analysis (Western blotting and immunofluorescence staining).

For the 10 day-infection, after 18 h of meningitis induction, the animals submitted to the behavioral test received ceftriaxone (100 mg/kg i.p. for 7 days) twice a day. Ten days after pneumococcal meningitis induction, nine animals from the sham group and nine from the meningitis group were submitted to the behavioral habituation test in the open field.

### Intracisternal injection of Evans blue-albumin.

Previous reports demonstrated that radiolabeled albumin and Evans blue-albumin (EBA), when injected into the brain parenchyma, migrate and concentrate in perivascular spaces, lymph vessels in the neck, and the cervical lymph nodes (61, 62). With fluorescent and colorimetric properties, Evans blue binds with affinity to albumin and has been used to assess the permeability of the BBB (63–65). The 1% solution of EBA was prepared to add 100 mg of Evans blue (Sigma-Aldrich, St. Louis, MO) and 100 mg of rat albumin (Sigma-Aldrich, St. Louis, MO) diluted in 10 mL of artificial cerebrospinal fluid (aCSF) (56). A 30G dental needle was inserted into one end of ca. 30-cm PE10 tubing and was connected to the other end of the tubing to a 100-μL Hamilton syringe. Then the cannula was filled with 30 μL of EBA solution. In sequence, the syringe was attached to the microinfusion pump. The anesthetized animal that received the bacterial suspension or aCSF was placed in a stereotaxic device on a heating pad, and the head was fixed on the apparatus with the rat's nose slightly pointed downwards. The cannula needle was inserted (approximately 1 to 2 mm) into the center of the cisterna magna, and the injection of EBA was started using the microinjection syringe pump at a rate of 1 μL/min for 25 min, resulting in a total volume injected of 25 μL of 1% EBA solution (66).

### Quantification of Evans blue-albumin in serum and brain.

At 4, 24, and 72 h after meningitis induction and EBA solution injection into the cisterna magna, blood was collected from the femoral artery and centrifuged for 10 min at 2,000 × *g* to isolate the serum. The rats were euthanized by decapitation under anesthesia, and the brains were placed in 3 mL of formamide (Sigma-Aldrich, St. Louis, MO) to allow EBA to diffuse from brain tissue and be held there for 72 h. The brains were removed, and the formamide was stored at −20°C until the spectrophotometry evaluation. CSF was not collected before the brains were harvested in order to maintain intact the CSF compartments for the EBA evaluation. The EBA concentration was measured in the serum and the formamide solution. Both samples were evaluated by spectrophotometry (620 nm) (67).

### Behavioral open-field tests.

Ten days after pneumococcal meningitis induction, nine animals from the sham group and nine animals from the meningitis group were submitted to the behavioral test of habituation to the open field. After the behavioral study, the rats were euthanized, and the brain was dissected for neurochemical evaluations. The open-field task evaluates motor performance in the training section and nonassociative memory in the retention test session. Habituation to an open field was carried out in a 40- by 60-cm open field surrounded by 50-cm high walls made of brown plywood with a frontal glass wall. The floor of the open field was divided into 9 equal rectangles by black lines. Each animal was gently placed on the left rear quadrant and left to explore the arena for 5 min (training session). Following this, the animals were taken back to their home cage, and 24 h later, they were submitted again to a similar open-field session (test session). The numbers of crossings (number of times the animal crossed the black lines, an assessment of locomotor activity) and rearing movements (exploratory behavior observed in rats subjected to a new environment) were counted in both sessions. The decrease in the numbers of crossings and rearings between the two sessions was taken as a measure of the retention of habituation memory (68). The test was conducted by a person who was blind to the groups.

### Coomassie staining and Western blot analysis.

Brains were homogenized in radioimmunoprecipitation assay (RIPA) buffer containing protease and phosphatase inhibitors using a cell strainer with a 100-μm-pore filter (Falcon). Brain homogenates, as well as CSF, serum samples, and purified Ply, and the liquid culture of type 3 S. pneumoniae at an optical density at 600 nm (OD_600_) of 0.5 were mixed with 1× LDS sample buffer (Thermo Fisher Scientific) and boiled at 95°C for 5 min. Protein samples (brain homogenates, CSF or serum samples, and purified proteins) were loaded into NuPage Novex 4 to 12% Bis-Tris SDS-PAGE gels (Thermo Fisher Scientific), electrophoresis was performed using Mini Gel Tank (Thermo Fisher Scientific), and electroblotting was performed on polyvinylidene difluoride (PVDF) membranes (Novex, Life Technologies) using the Mini Blot Module (Thermo Fisher Scientific). Ply, NSE, Iba1, IFN-γ, TMEM119, and AQP4 were detected using, respectively, a mouse anti-Ply antibody (Abcam), a rabbit anti-NSE antibody (Merck), a goat anti-Iba1antibody (Abcam), a rabbit anti-IFN-γ antibody (Abcam), a rabbit anti-TMEM119 antibody (Thermo Fisher), and a rabbit anti-AQP4 antibody (Abcam). All primary antibodies were diluted 1:1,000 in PBS–0.1% Tween (PBST); the secondary antibodies used were horseradish peroxidase (HRP)-conjugated goat anti-mouse, goat anti-rabbit, and donkey anti-goat (Thermo Fisher Scientific), all diluted 1:5,000 in PBST. Protein bands were detected by incubating membranes with ECL Prime Western blotting detection reagents (Cytiva, Thermo Fisher Scientific) and imaged using ImageQuant LAS 4000 (GE Healthcare). For Coomassie staining, after electrophoresis gels were incubated with InstantBlue Coomassie protein stain (Abcam) for 15 min and then photographed. The intensity of protein bands on PVDF membranes was measured by ImageJ, as previously described (69). Percentages of protein expression were calculated as a ratio of the protein band intensity divided by the total protein content (after Coomassie staining) measured by ImageJ in each specific lane of the gel/membrane: the same volume of protein lysate was loaded onto gels for Coomassie staining to perform the quantification of the total protein content in each sample and on a separate gel for protein detection by Western blotting.

### Immunofluorescence staining.

At 4, 24, and 72 h after meningitis induction, the animals were anesthetized with ketamine (6.6 mg/kg) and xylazine (0.3 mg/kg). Then, the CSF samples were collected through a puncture of the cisterna magna. After the animals were perfused transcardially with PBS and euthanized by decapitation, the brains were removed, immediately soaked in Cryomatrix (Thermo Fisher Scientific), and then stored in a −80°C freezer. Coronal sections of 20-μm thickness of frozen rat brains embedded in Cryomatrix (Thermo Fisher Scientific) were cut and collected on Superfrost Plus slides (Thermo Fisher Scientific). Sections were fixed with acetone for 10 min and dried, and the edges of the sections were marked with a PAP pen (Avantor, VWR) and incubated with one of the following combinations: (i) rabbit polyclonal anti-capsule serotype 3 antiserum (SSI Diagnostica) followed by Alexa Fluor 594-conjugated goat anti-rabbit (Thermo Fisher Scientific), (ii) mouse anti-MAP2 antibody combined with chicken anti-Tau antibody (Abcam) followed by Alexa Fluor 594-conjugated goat anti-mouse (Thermo Fisher Scientific) and Alexa Fluor 594-conjugated goat anti-chicken (Thermo Fisher Scientific), or (iii) mouse anti-GFAP antibody (Santa Cruz Biotechnology) followed by Alexa Fluor 488-conjugated goat anti-mouse (Thermo Fisher Scientific) combined with rabbit anti-AQP4 antibody followed by Alexa Fluor 647-conjugated goat anti-rabbit (Thermo Fisher Scientific) mixed with Lycopersicon esculentum tomato lectin DyLight 594 (Vector Laboratories). Incubations with primary antibodies were performed for 2 h at room temperature, and incubations with secondary antibodies were performed for 2 h at room temperature in the dark. The dilution range of primary antibodies was 1:50 to 1:200, and the dilution range of secondary antibodies was 1:500 to 1:1,000; both primary and secondary antibodies were diluted using PBS–1% bovine serum albumin (BSA).

For staining of CSF samples, drops of 10 μL were pipetted onto microscope glass slides and left to dry at room temperature; the edges of each drop were marked with a PAP pen and incubated with rabbit polyclonal anti-capsule serotype 3 antiserum (SSI Diagnostica) for 1 h at room temperature, followed by Alexa Fluor 594-conjugated goat anti-rabbit (Thermo Fisher Scientific) for 1 h at room temperature in the dark.

Before starting the staining procedure, all samples were blocked with PBS with 5% BSA (Sigma-Aldrich) for 15 min at room temperature. All antibodies were diluted according to the recommendations from the manufacturers using PBS with 1% BSA, and slides were washed in PBS three times for 5 min between primary and secondary antibody incubations. Slides were finally mounted using ProLong Diamond antifade mountant (Invitrogen, Thermo Fisher Scientific).

### Microscopy analysis.

All stained samples were imaged using a Zeiss Observer.Z1 fluorescence microscope with an Orca-Flash4.OLT camera. To acquire the images of the entire CSF, 10-μL drops of the brain tissue sections were examined with a 5× objective combined with the function “Tiles” of the imaging program Zen 2 (Zeiss). For imaging with higher magnification of BBB vascular endothelium (tomato lectin) and astrocytes (anti-GFAP antibody followed by Alexa Fluor 488-conjugated goat anti-mouse), a 63× objective combined with the function “Stacks” of the imaging program Zen 2 was used; each image taken was a merge of 15 to 20 stacks imaged. Confocal microscopy analysis was performed using a Zeiss LSM980-Airy2 confocal system. To acquire images, the program ZEN lite (Zeiss) was used, while 3D modeling after image acquisition was performed using Imaris (Oxford Instruments). During the confocal microscopy imaging, each z-stack image included 15 stacks with a total thickness of 15 μm. For the microscopy analysis with high-magnification objectives (both ordinary fluorescence and confocal microscopy), we decided to focus on the septum, in the tissue between the area underneath the subarachnoid space and the more external part of the cerebral cortex.

### Quantification of the fluorescence signal and colocalization analysis.

As previously described, fluorescence signal poststaining was quantified using the software Image J (42). Briefly, all fluorescence images were converted into grayscale images, and then using the function “Threshold,” the surface covered by the fluorescence signal was defined with the function “Create Selection” and finally quantified using the function “Measure.” For colocalization analysis, fluorescence signals from tomato lectin and astrocytes (stained with anti-GFAP antibody) were analyzed for colocalization using the “Co-localization” plugin, which turns all of the colocalized signals into a white color; the surface covered by the colocalized white color was then measured using the function “Measure.”

### Statistical analysis.

For statistical analysis related to the quantification of Evans blue-albumin in Wistar rats, the software SPSS was used; for the statistical analysis related to all other *ex vivo* analyses, the software Graph Pad (Prism 5) was used. For all multiple comparisons, nonparametric analysis of variance (ANOVA) was used to assess the presence of differences between the groups, and then a Dunn's test was applied for pairwise comparisons. For 2-group comparisons, the nonparametric 2-tailed Wilcoxon's rank sum test (also known as the Mann-Whitney *U* test) was used. *P* values are indicated in each figure legend.

### Data availability.

The data sets used and/or analyzed during the current study are available from the corresponding author on reasonable request.
