# Supplementary figures and images for: Dysfunctional Glymphatic System with Disrupted Aquaporin 4 Expression Pattern on Astrocytes Causes Bacterial Product Accumulation in the CSF during Pneumococcal Meningitis
Source: mBio. 2022 Aug 29;13(5):e01886-22. doi: 10.1128/mbio.01886-22 (PMC9600563; doi:10.1128/mbio.01886-22)

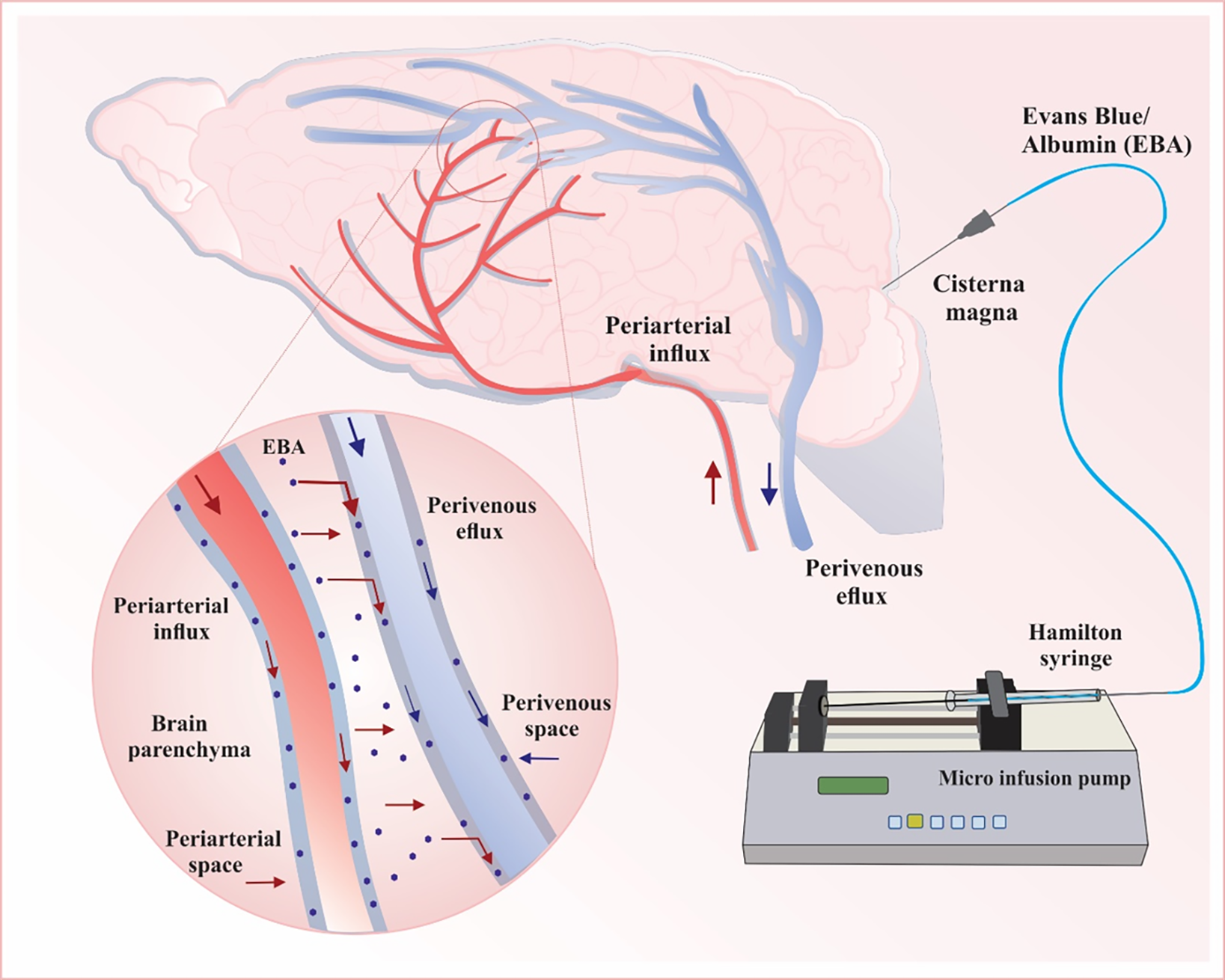

Supplement: FIG S1 [file mbio.01886-22-s0001.tif]

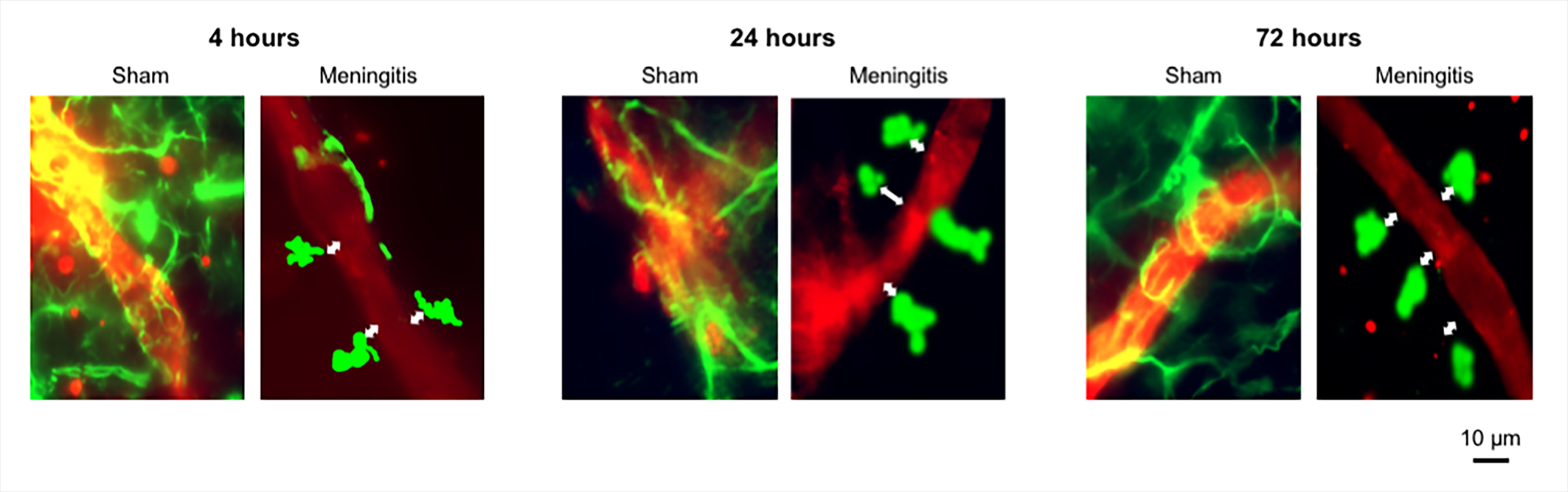

Supplement: FIG S2 [file mbio.01886-22-s0002.tif]

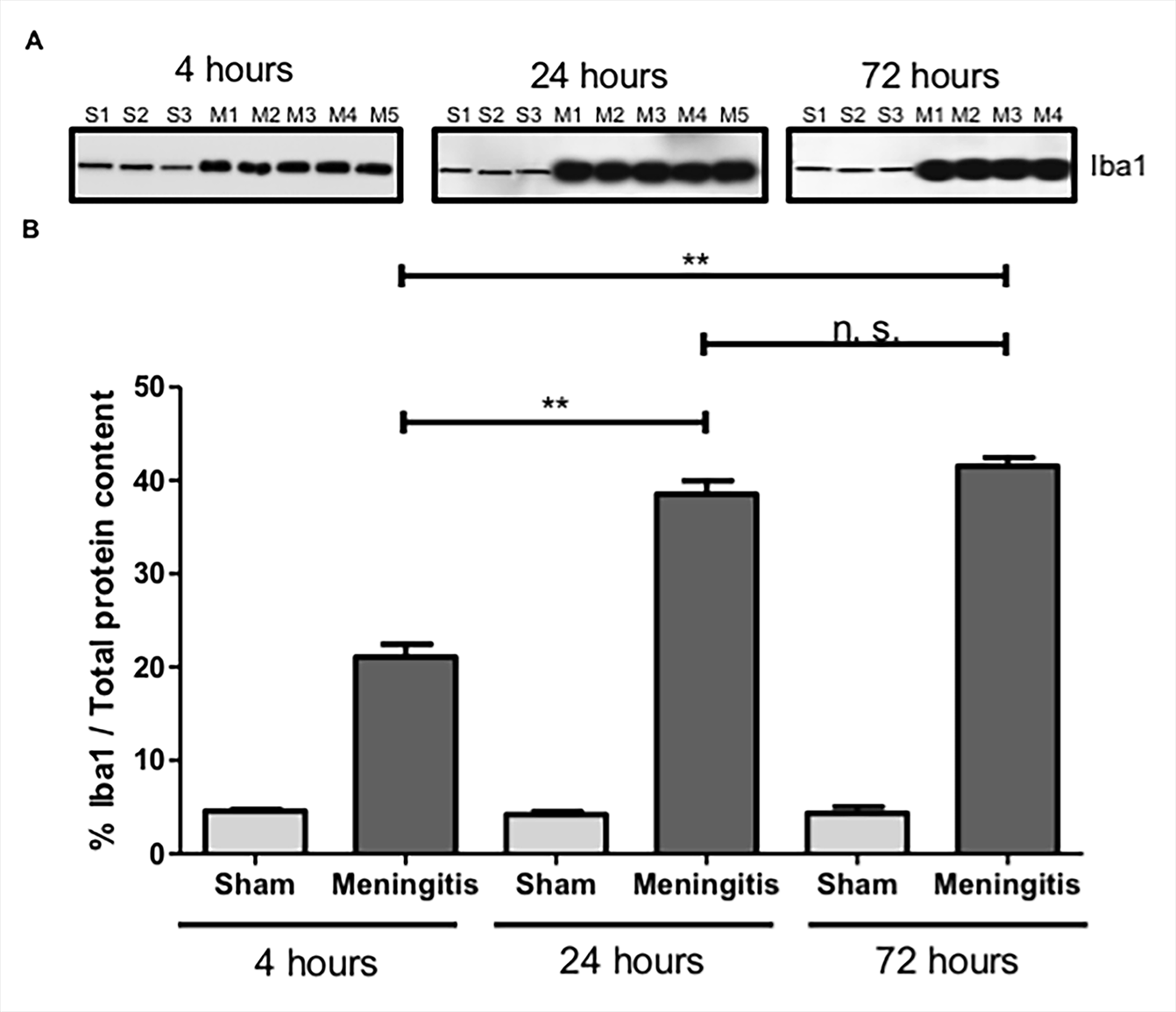

Supplement: FIG S5 [file mbio.01886-22-s0005.tif]

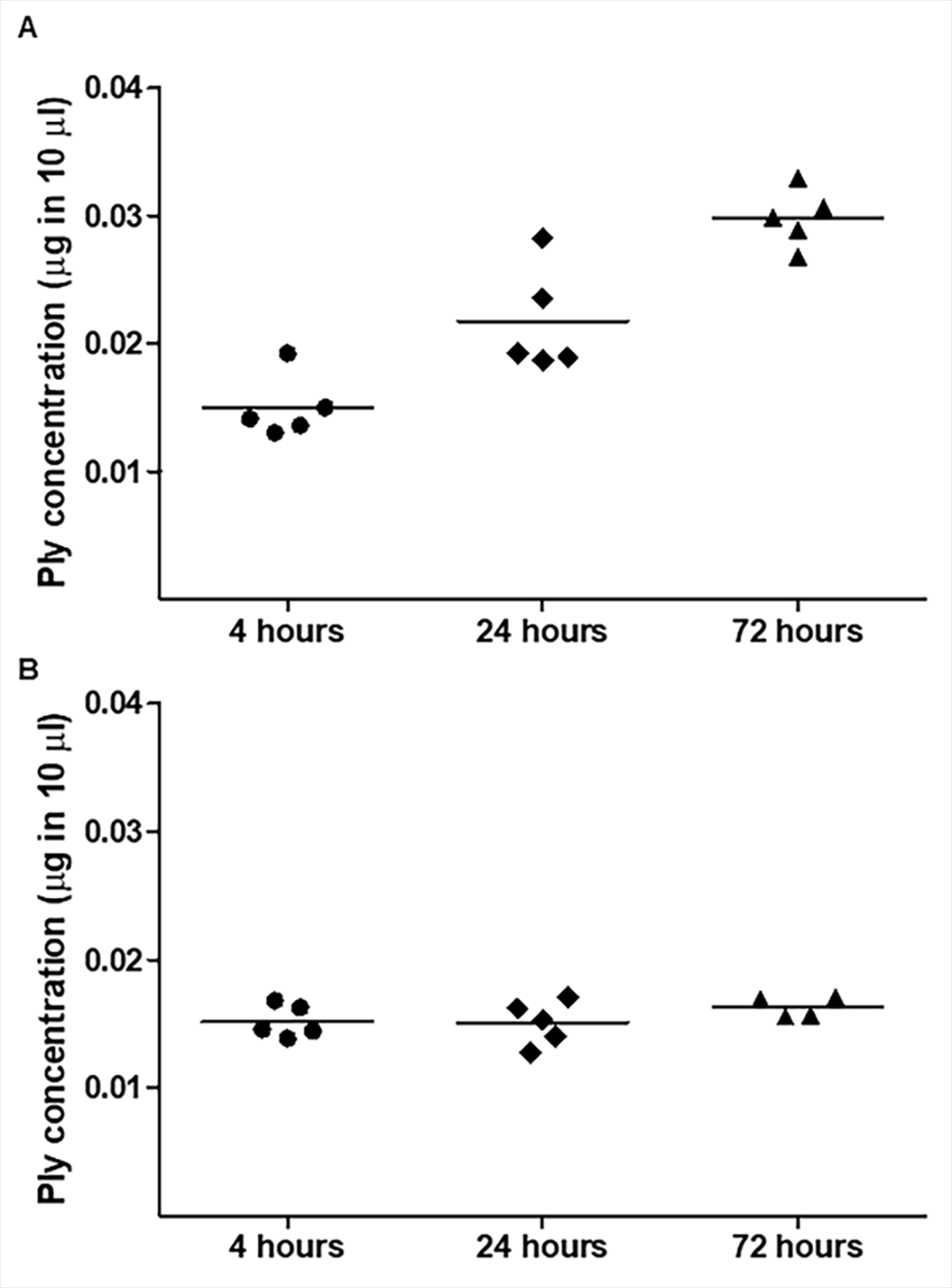

Supplement: FIG S3 [file mbio.01886-22-s0003.tif]

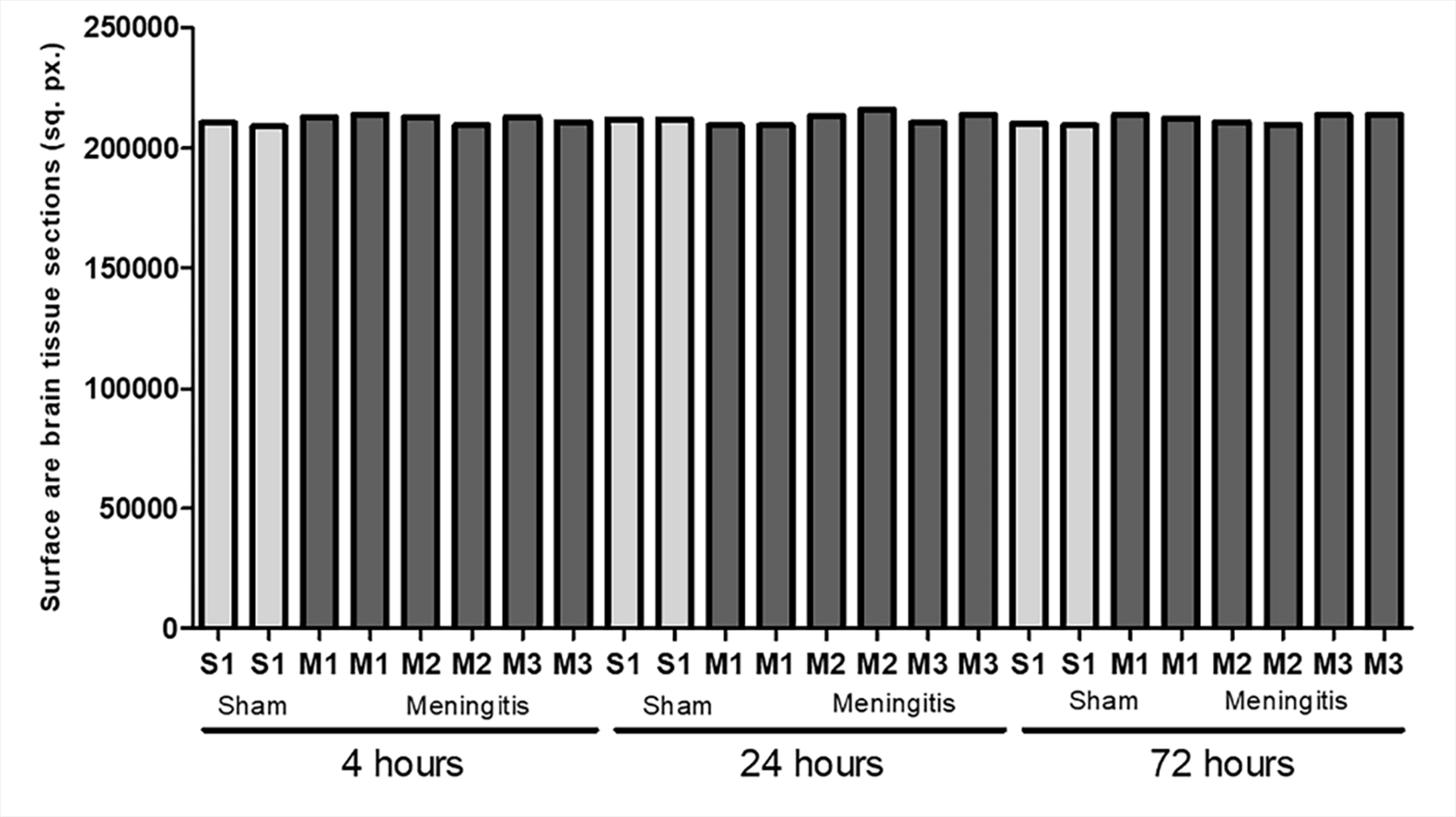

Supplement: FIG S4 [file mbio.01886-22-s0004.tif]

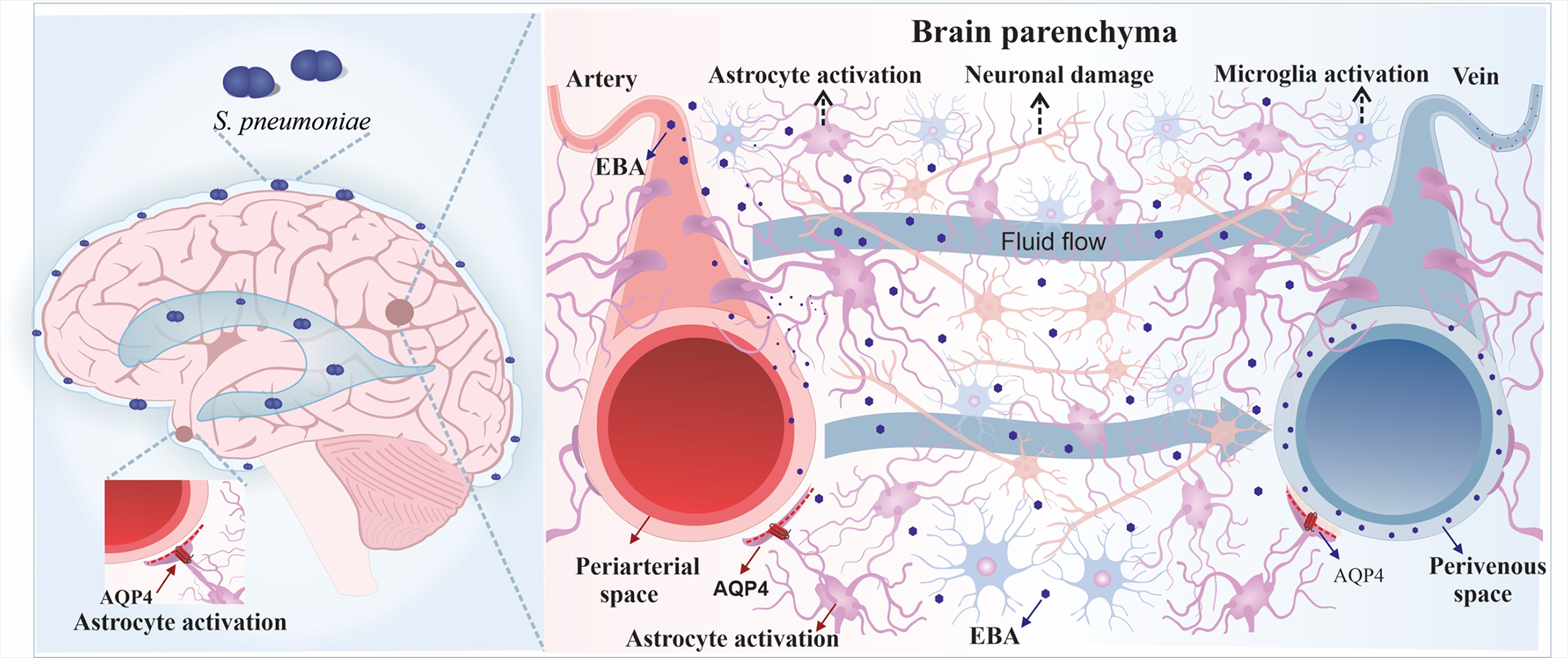

Supplement: FIG S6 [file mbio.01886-22-s0006.tif]
