# Supplementary material for: Dysfunctional Glymphatic System with Disrupted Aquaporin 4 Expression Pattern on Astrocytes Causes Bacterial Product Accumulation in the CSF during Pneumococcal Meningitis
Source: mBio. 2022 Aug 29;13(5):e01886-22. doi: 10.1128/mbio.01886-22 (PMC9600563; doi:10.1128/mbio.01886-22)
Supplement: TABLE S1 [file mbio.01886-22-s0007.docx]

| Time | 1 = Coma | 2 = Does not turn upright when positioned on the back | 3 = Turns upright within 30 s | 4 = Minimal ambulatory activity, turns upright < 5 s | 5 = Normal | Eye secretion | Coat (piloerection) | Weight | CFU/mL in CSF (5 µl of CSF) |
| --- | --- | --- | --- | --- | --- | --- | --- | --- | --- |
| 4 hours |  |  |  |  | R1  R2  R3  R4  R5 | R1 |  | R1 - 210 g  R2 - 222 g  R3 - 215 g  R4 - 219 g  R5 - 217 g | R1 4.3x10^4^  R2 1.5x10^5^  R3 1.1x10^5^  R4 6.6x10^4^  R5 5.4x10^4^  **AV 8.5x10^4^** |
| 24 hours |  |  |  | R1  R2  R3  R4  R5 |  |  | R1  R2  R3  R4 | R1 - 221 g  R2 - 215 g  R3 - 215 g  R4 - 227 g  R5 - 212 g | R1 2.0x10^5^  R2 7.4x10^5^  R3 6.5x10^5^  R4 6.8x10^5^  R5 8.4 x10^4^  **AV 4.7x10^5^** |
| 72 hours |  |  |  | R1  R2  R3  R4  R5 |  |  | R1  R2  R3  R4  R5 | R1 - 210 g  R2 - 215 g  R3 - 215 g  R4 - 220 g  R5 - 218 g | R1 6.9x10^5^  R2 7.7x10^5^  R3 8.5x10^5^  R4 6.7x10^5^  R5 1.8x10^6^  **AV 9.6x10^5^** |
